# Expanding landscapes of the diversified *mcr-1*-bearing plasmid reservoirs

**DOI:** 10.1186/s40168-017-0288-0

**Published:** 2017-07-06

**Authors:** Qingjing Wang, Jian Sun, Jun Li, Youfa Ding, Xing-Ping Li, Jingxia Lin, Bachar Hassan, Youjun Feng

**Affiliations:** 1 Department of Medical Microbiology and Parasitology, Zhejiang University School of Medicine, Hangzhou, 310058 Zhejiang China; 2 National Risk Assessment Laboratory for Antimicrobial Resistance of Animal Original Bacteria, College of Veterinary Medicine, South China Agricultural University, Guangzhou, 510642 Guangdong China; 3 Key Laboratory of Bioorganic Synthesis of Zhejiang Province, College of Biotechnology and Bioengineering, Zhejiang University of Technology, Hangzhou, 310014 Zhejiang China; 4 Lishui People’s Hospital, Lishui, 323000 Zhejiang China; 5 University of North Carolina, Chapel Hill, NC USA

**Keywords:** Landscape, Plasmid genome, *mcr-1*, Colistin resistance, Dissemination

## Abstract

**Background:**

Polymyxin is a cationic polypeptide antibiotic that can disrupt bacterial cell membrane by interacting with its lipopolysaccharide molecules and is used as a last resort drug against lethal infections by the carbapenem-resistant superbugs (like NDM-1). However, global discovery of the MCR-1 colistin resistance dramatically challenges the newly renewed interest in colistin for clinical use.

**Methods:**

The *mcr-1*-harboring plasmids were acquired from swine and human *Escherichia coli* isolated in China, from 2015 to 2016, and subjected to Illumina PacBio RSII and Hi-Seq2000 for full genome sequencing. PCR was applied to close the gap of the assembled contigs. Ori-Finder was employed to predict the replication origin (*ori*C) in plasmids. The phenotype of MCR-1-producing isolates was evaluated on the LBA plates with various level of colistin. Genetic deletion was used to test the requirement of the initial “ATG” codon for the MCR-1 function.

**Results:**

Here, we report full genomes of over 10 *mcr-1*-harboring plasmids with diversified replication incompatibilities. A novel hybrid IncI2/IncFIB plasmid pGD17-2 was discovered and characterized from a swine isolate with colistin resistance. Intriguingly, co-occurrence of two unique *mcr-1*-bearing plasmids (pGD65-3, IncI2, and pGD65-5, IncX4) was detected in a single isolate GD65, which might accelerate dissemination of the *mcr-1* under environmental selection pressure. Genetic analyses of these plasmids mapped mobile elements in the context of antibiotic resistance and determined two insertion sequences (IS*Ecp1* and IS*Apl1*) that are responsible for the mobilization of *mcr-1*. Gene deletion also proved that the first ATG codon is redundant in the *mcr-1* gene.

**Conclusions:**

Collectively, our results extend landscapes of the diversified *mcr-1*-bearing plasmid reservoirs.

**Electronic supplementary material:**

The online version of this article (doi:10.1186/s40168-017-0288-0) contains supplementary material, which is available to authorized users.

## Background

Antibiotics play crucial roles in controlling the dissemination of lethal infections by bacterial pathogens. However, the emergence of antibiotic resistance is developing a great threat to public health worldwide. It is estimated that multidrug-resistant (MDR) pathogens cause nearly 700,000 cases of lethal infections (including 214,000 neonatal deaths) each year [1]. The discovery of New Delhi β-lactamase 1 (NDM-1) and its variants like NDM-5 had ever pushed us on the cusp of post-antibiotic era, in that they can confer potent resistance to extended spectrum β-lactamases (ESBL) and carbapenems, the two classes of widely used antibiotics against the MDR bacteria [2, 3]. In general, the cationic polypeptide antibiotic polymyxin E (colistin) is believed to act as a last resort against fatal infections by gram-negative pathogens with pan-drug resistance [4, 5]. However, it seems likely that gut bacteria have evolved some mechanisms to bypass the final line of refuge antibiotic (i.e., colistin resistance) [4–7]. Biochemical mechanism by which bacteria acquire the appreciable resistance to colistin is mainly dependent on the reduction in the net negative charge of the bacterial outer-membrane, which consequently decreases the binding affinity of colistin to bacterial surface [8–10]. To the best of our knowledge, four types of modifications of the lipid A moiety anchored on bacterial lipid polysaccharide (LPS) are involved in the alteration of the net negative surface charge, which include (i) modification at 3′-linked second fatty acyl chain with glycine (and/or diglycine) [8]; (ii) the addition of amino-arabinose to 4′-phosphate position of sugar [9]; (iii) the addition of sugar with phosphoethanolamine (pEtN) at 4′-phosphate position [10, 11]; and (iv) the attachment of galactosamine (GalN) to 1′-phosphate of sugar [12, 13]. Unlike the fact that a single ArnT enzyme catalyzes the reaction for amino-arabinose-based sugar modification of lipid A in *Pseudomonas aeruginosa* (*P. aeruginosa*) [14] and *Cupriavidus metallidurans* [9], a three-component system (AlmG-AlmF-AlmE, encoded by an operon of Vc1577-Vc1578-Vc1579) is evolved to modify the lipid A with glycine in *Vibrio cholerae* [8]. The pEtN modification of lipid A originally found in *Klebsiella pneumoniae* (*K. pneumoniae*) is attributed to the point mutations of the *phoP-phoQ* two-component system and its regulator gene *mgrB* [11]. Intriguingly, the modifications of lipid A with both GalN and pEtN account for colistin resistance in the serious opportunistic pathogens *Acinetobacter baumannii* (*A. baumannii*) [13, 15]. Additionally, the PmrA/PmrB two-component system is also implicated into genetic control of lipid A modification as well as polymyxin resistance in *Salmonella enterica* (*S. enterica*) [16], *A. baumannii*, and *P. aeruginosa* [14]. Thus, we anticipated that enteric pathogens have developed multiple mechanisms to remodel its lipid A structure/integrity on the LPS under dynamic selection by the altered environments, like antibiotic exposures.

The emergence of the mobilized colistin resistance gene (*mcr-1*) illustrated a new mechanism for plasmid-borne polymyxin resistance [6]. The protein product of *mcr-1*, referred to MCR-1, is annotated as an integral membrane protein with five trans-membrane helices [7]. Our genetic experiment proved that localization of the MCR-1 in bacterial peri-plasm is critical for its role in colistin resistance through deleting the trans-membrane region [7]. Together with catalytic structures of MCR-1 reported by three other research groups [17–19], our structure-guided functional studies revealed the requirement of a five-residue-containing motif for its biochemical function [7]. Since its first discovery in Southern China in the late of 2015 [6], the *mcr-1* gene have been detected in nearly 40 countries from five of seven continents [7, 20]. The *mcr-1*-positive gut bacteria included *Escherichia coli* (*E. coli*) [21, 22], *S. enterica* [23, 24], *K. pneumoniae* [6, 25, 26], *Enterobacter aerogenes* (*E. aerogenes*) [27], *Kluyvera ascorbata* [28], *Citrobacter freundii* [29], and *Citrobacter braakii* [30]. Not only does the *mcr-1* gene appear in diversified *E. coli* isolates with different sequence types [20, 31], but also it coexists with the NDM-1 [32, 33] and its variants, like NDM-5 [34, 35] and NDM-9 [36]. Additionally, ESBL can be coproduced with MCR-1 from a single plasmid or a same bacterial isolate [37–39]. Further sequence analyses showed that the *mcr-1-*harboring plasmid reservoirs exhibit an unexpected diversity [20–22]. Multiple lines of replication incompatibility have been assigned to these *mcr-1*-bearing plasmids, which included IncP [26], IncI2 [6, 23, 24, 35, 40, 41], IncX1-X2 mosaic version [42], IncX3-X4 hybrid type [43], IncX4 [7, 44, 45], IncHI2 [23, 24, 35, 46], IncFI [47], IncFII [48], and IncHI1 [49], respectively. Obviously, it seems likely that the spread of *mcr-1* proceeds by complex dissemination across diversified species among Enterobacteriaceae.

Our recent study suggested that a group of *mcr-1*-positive plasmids with distinct backbones are present in swine farm *E. coli* isolates as well as human clinical *E. coli* isolates [20]. Here, we report comparative genomics of over 10 representative *mcr-1*-bearing plasmids. Unexpectedly, we identified a new *mcr-1*-carrying hybrid plasmid pGD17-2 classified into two different replication incompatibilities (IncFIB and IncI2). Moreover, we are first to observe coexistence of two unique *mcr-1*-positive plasmids (IncI2-type pGD65-3 and IncX4-like pGD65-4) in a same *E. coli* isolate. In addition, we determined a couple of variants of the other known *mcr-1*-positive plasmids. These findings expand greatly the landscape of *mcr-1*-harboring plasmids.

## Methods

### Conjugation, S1-PFGE, and Southern hybridization

The *mcr-1*-positive samples we obtained were kept in LB broth or on LB agar with appropriate antibiotics. *E. coli* C600 was used as the recipient for the conjugation experiment of MCR-producing *E. coli* isolates. The trans-conjugants were selected on MacConkey agar containing colistin (2 μg/ml) and streptomycin (2000 μg/ml) and finally confirmed by PCR and ERIC-PCR. To analyze the location of the *mcr-1* gene, *S1*-PFGE and Southern blot analysis were performed using the original strains and trans-conjugants carrying *mcr-1* gene. Briefly, whole-cell DNA of the *E. coli* strains were extracted and embedded in agarose gel plugs. Subsequently, the agarose gel plugs were treated with *S1* nuclease (TaKaRa, Dalian, China), and the DNA fragments were separated by PFGE. Southern blot hybridization was then performed with the digoxigenin-labeled probes (Roche Diagnostics, Mannheim, Germany) specific for the *mcr-1* gene.

### Bacterial samples, plasmid sequencing, and sequence assembly

The plasmids and primers used in this study are listed in Additional file 1: Tables S1 and S2. Fourteen plasmids were sequenced by Illumina PacBio RSII and Hi-Seq2000 using prepared paired-end 2 × 200-bp libraries, of which raw data were separately assembled using SOAP de novo (version 2.0.4) and SMRT Analysis (version 2.2.0), after high-quality read acquisition, and any assembly discrepancies/uncertainties filled or closed with PCR. The finally generated contigs were checked for circularization, whereas overlapping ends were trimmed. The general features of the 14 plasmids are summarized in Additional file 1: Table S1. Ori-Finder was employed to predict the replication origin (*ori*C) in plasmids under study [50]. The assembled plasmid sequences were annotated by using the RAST suite for the identification of protein-coding sequences, and ARAGORN [51] for tRNA, and RNAmmer [52] for rRNA.

### Annotation of plasmids and resistance genes

A bacterial mobilome annotation procedure [53] was designed to type sequenced/assembled plasmids and to identify insertion sequences (ISs), integrons, and prophages. The *mcr-1* gene has been proposed to confer colistin resistance, while other mobile-related elements were detected using specific informatics tools. PlasmidFinder [54] was used to type replicons of plasmids that had been previously assembled and identified. ISs were mainly identified with ISsaga [55] and ISfinder [56] and then manually adjusted with focuses on intactness, terminal inverse repeats (IRs), and flanking direct repeats (DRs). IS*CR* elements were also identified (http://www.cardiff.ac.uk/research/explore/research-units/antibacterial-agents-and-genetics-of-resistance). Prophages were predicted with PHAST [57] and *Phage_Finder* [58]. Resistance genes were in silico identified with ARDB [59], CARD [60], and ResFinder [61].

### Determination of colistin resistance

According to the recommendation of EUCAST, micro-broth dilution method was used to address colistin MIC, whose level of ≤0.25 μg/ml was treated susceptible. For functional evaluation of *mcr-1* derivatives in colistin resistance, agar dilution method was applied and resistance level of the colistin-susceptible strain MG1655 (≤2.0 μg/ml) is the cutoff value.

### Nucleotide sequence accession number

The plasmid nucleotide sequences reported in this study have been deposited in GenBank under accession number listed in Additional file 1: Table S1.

## Results and discussion

### Genetic evidence for the presence of *mcr-1* in diversified plasmids

Our recent study reported hundreds of *mcr-1*-positive plasmids through extensive dissection of a thousand of *E. coli* isolates from swine farm and hospitalized patients, which implied that the diversified *mcr-1*-carrying plasmids are present [20]. In this follow-up study, 13 representative *mcr-1*-positive plasmids (Additional file 1: Table S1) were subjected to further genetic analyses of both *S1*-pulsed-field gel electrophoresis (PFGE) (Additional file 2: Figure S1A and C) and Southern hybridization blot (Additional file 2: Figure S1B and D). As expected, diversified plasmids were present in the above 13 *E. coli* isolates (Additional file 2: Figure S1). Of note, ten trans-conjugants were successfully obtained from the 13 *mcr-1*-positive isolates. Southern blotting revealed that the *mcr-1* gene was present on plasmids ranging from ~33 to 240 kb (Additional file 2: Figure S1). First, it was estimated that no less than three isolates (GD46, GD65, and WH03) carries the *mcr-1*-positive plasmid of ~33 kb long (Additional file 2: Figure S1B and D). Second, the *mcr-1* gene is located on ~60 kb plasmid in seven isolates (Additional file 2: Figure S1B and D). Third, two big *mcr-1*-harboring plasmids (~120 kb for pGD17-2 and ~240 kb for pGD80-2) separately occurred in the strains GD17-2 and GD80-2 (Additional file 2: Figure S1B and D). Although we failed to detect *mcr-1*-positive band in the strain Lishui12 using Southern blot (Additional file 2: Figure S1B), we proved that it is PCR-positive for *mcr-1*, and had a success in isolating a *mcr-1*-producing plasmid pLishui12 of ~33 kb long (Additional file 2: Figure S4). Curiously, in the strain GD65, the *mcr-1* gene was found on two distinct plasmids with different sizes (~30 kb for pGD65-4 and ~60 kb for pGD65-3, Additional file 2: Figure S1B). The physical evidence for the presence of *mcr-1* in the above plasmids was validated by further PCR analyses combined with direct DNA sequencing. All the above *mcr-1*-positive plasmids can confer the *E. coli* strains to possess modest level of colistin MIC [2–4 μg/ml] (Table 1).

**Table 1:** Colistin MIC of clinical *E. coli* strains expressing *mcr-1*

	Strains												
Colistin MIC (μg/ml)	GD17	GD65	GD80	GD23	GD53	LS142	WH07	WH09	WH13	GD81	LS12	GD46	WH03
	2	4	4	2	2	4	4	4	4	2	4	4	4

### Overall landscape of the *mcr-1*-carrying plasmids

**Fig. 1:**
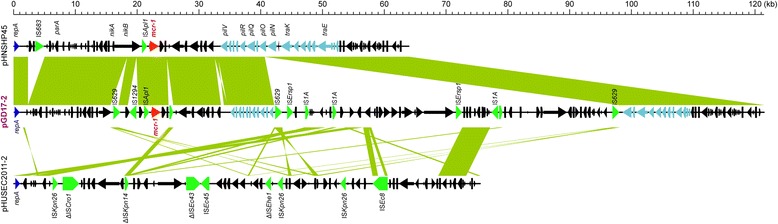
Discovery of a new hybrid plasmid pGD17-2 that comprises the *mcr-1* gene. The full genome of pGD17-2 is sequenced and collinearly compared with the counterpart of pHNSHP45 and pHUSEC2011-2. The mosaic plasmid pGD17-2 (highlighted in purple) is isolated from a colistin-resistant strain GD17. The *mcr-1*-positive IncI2-type plasmid pHNSHP45 and the *mcr-1*-negative plasmid pHUSEC2011-2 both are applied to define the plasmid backbone of pGD17-2, giving the presence of two incompatible replicon types (IncFIB and IncI2). The relevant parts of plasmid are also shown to highlight the syntenic regions. Regions of synteny between adjacent schematics are indicated by the *shaded areas*. These schematics are drawn to scale. Genes associated with the *tra* and *pil* loci are indicated by *light blue arrows*, while replication-associated genes are denoted as *dark blue arrows*. The *mcr-1* gene is indicated with the *red arrow*, while accessory genes are indicated by *black arrows*. Insertion sequences are highlighted in *green arrows*

**Fig. 2:**
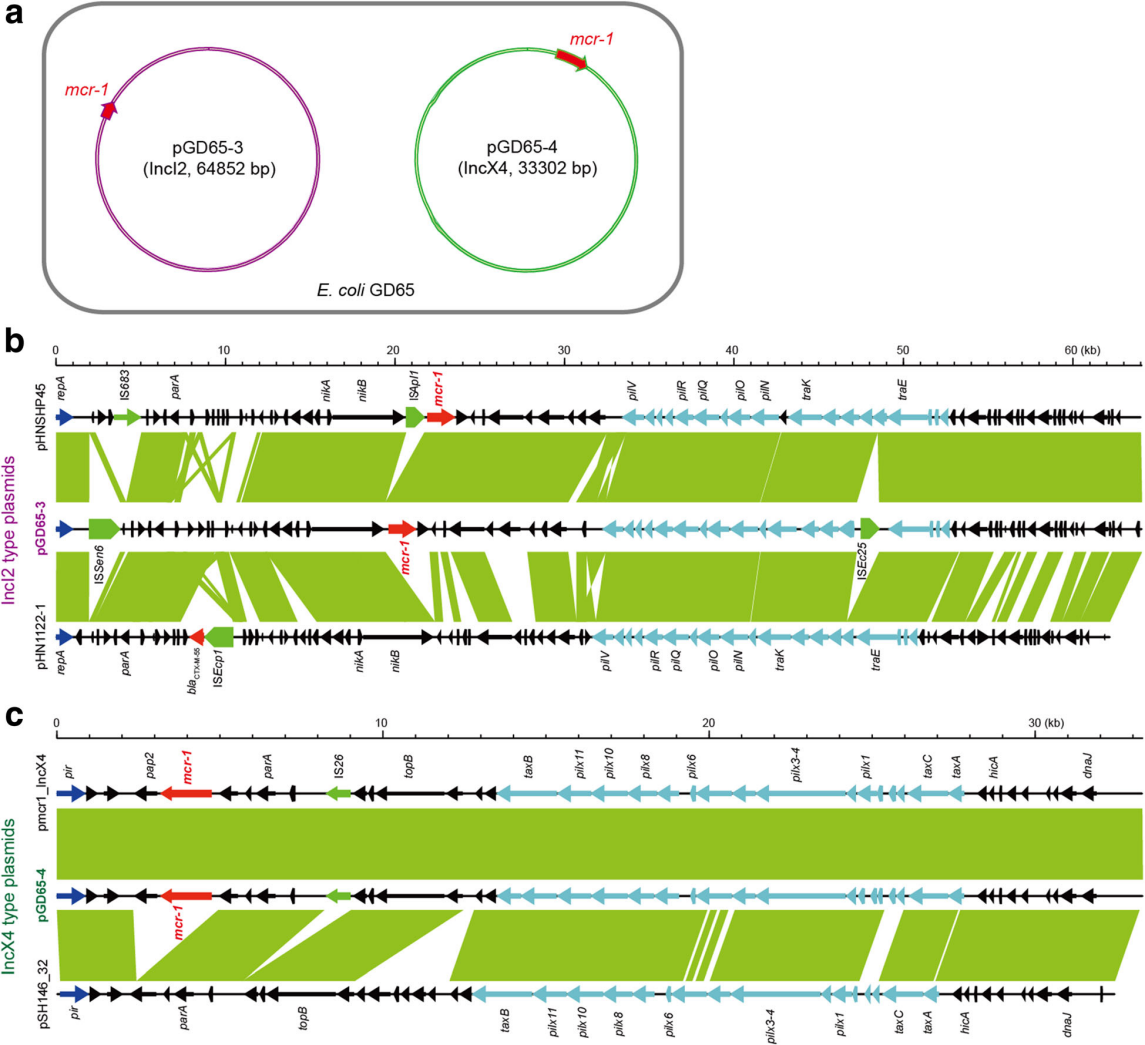
Co-existence of two distinct incompatible *mcr-1*-bearing plasmids (pGD65-3 and pGD65-4) in a single colistin-resistant *E. coli* isolate GD65. **a** Cartoon diagram for the two *mcr-1*-positive plasmids (pGD65-3 and pGD65-4) that coexist in an *E. coli* isolate GD65. **b** Linear comparison of the IncI2-type plasmid pGD65-3 with other two closely related *mcr-1*-harboring plasmids pHNSHP45 and pHN1122-1. **c** Linear genome alignments of the IncX4-type plasmid pGD65-4 with other two closely related *mcr-1*-bearing plasmids pMCR1_IncX4 and pSH146_32. Regions (>99% similarity) are marked by green shading. Genes associated with the *tra* and *pil* loci are separately indicated by *light blue arrows*, while replication-associated genes are denoted as *dark blue arrows*. The *mcr-1* genes are highlighted in *red*, while insertion sequences are in *green arrows*

In total, full replicon sequences of 13 *mcr-1*-harboring plasmids were assembled through Hi-Seq sequencing (Illumina Inc.), in which the gaps were closed with ABI 3730 Sanger sequencing (Additional file 1: Table S1). Using specific primers (Additional file 1: Table S2), all these plasmids were confirmed by the close-loop PCR (Additional file 2: Figure S8A). BLAST-based query using PlasmidFinder (https://cge.cbs.dtu.dk/services/PlasmidFinder/) revealed that the newly-determined 14 plasmids are classified into four types as follows: eight IncI2-type plasmids, four IncX4-like plasmids, one IncHI2-type, and one IncI2/IncFIB hybrid plasmid pGD17-2 (Additional file 1: Table S1 and Additional file 2: Figures S1-S7). It is noted that (i) the insertion sequence IS*Apl1* is located immediately upstream of the *mcr-1* gene (Figs. 1 and 2) and (ii) a putative locus *pap2* encoding the PAP2-family protein is neighbored with the downstream of the *mcr-1* gene (Fig. 2), which might facilitate the transfer of *mcr-1* [39]. We also noted that the insertion sequence of IS*Apl1* is lacking in some cases, implying a relic for frequent conjugation of IS*Apl1-mcr-1-pap2*. Therefore, the discovery of these plasmids significantly contributed to a growing collection of *mcr-1*-positive plasmids with known replicon sequences (Additional file 1: Table S3).

Briefly, in similarity to the newly identified plasmid pLishui142-1 (61.908 kb), seven additional new members of IncI2-type *mcr-1*-bearing plasmids were determined in this study, which corresponded to pGD23-3 (64.078 kb), pGD53-3 (62.813 kb), pGD65-3 (64.857 kb), pGD81-1 (62.712 kb), pWH07-3 (62.067 kb), pWH09-3 (62.072 kb), and pWH13-4 (60.694 kb), respectively (Additional file 1: Table S1). Although this type of plasmids lacks the insertion sequence IS*Apl1* (Additional file 2: Figure S2), they consistently share a site-specific recombinase (*rci*) gene, two pilus-encoding genes (*pil* and *tra*), and four different pilli proteins (Additional file 2: Figure S2). In addition, we found that heterogeneity of whole-shufflon structural variations exists between the 3′ region of *pilV* gene and *rci* gene in *mcr-1*-harboring IncI2 plasmids as recently reported [62]. The plasmid sequences yielded the following results: in pWH9-3 and pWH7-3, *rci*-A-A′-C-C′-B′-D′-*pilV*; in pWH13-4, *rci*-A-A′-C-C′-D′-B′-*pilV*; in LS142-1, *rci*-B′-D′-A-A′-*pilV*; in 65-3, *rci*-A′-A-D′-B′-C-C′-B′-D′-A-A′-*pilV*; in pGD23-3, *rci*-C-C′-D′-B′-A′-A-B′-D′-*pilV*; in pGD53-3, *rci*-C-C′- B′-D′-D′-B′-A′-A-*pilV*; and in hybrid plasmid pGD17-2, *rci*-C-C′-A-A′-D′-B′-*pilV*. Unlike the scenario seen with the IncHI2 plasmid pHNSHP45-2 that carries the *mcr-1* gene [46], the typical *mcr-1* cassette is located in different locations of pGD80-2 (241.033 kb), a member of the IncHI2-type plasmid family (Additional file 2: Figure S3). Moreover, it encodes three more resistance genes (*aph*(*3*′)*-Ia, oqxAB*, and *tetR*), suggesting complexity/flexibility among the *mcr-1*-bearing IncHI2 plasmids.

In addition to pLishui12 (33.309 kb) [63], three more *mcr-1-*harboring IncX4 plasmids we reported here included pGD46-3 (33.302 kb), pGD65-4 (33.305 kb), and pWH03 (33.292 kb), respectively (Additional file 1: Table S1). All the four IncX4 plasmids are featuring the almost identical genetic backbone in which no IS*Apl1* element is flanked by the only antibiotic resistance gene *mcr-1* gene (Additional file 2: Figures S4, S5, and S7). Genome alignments indicated that plasmid pGD46-3 was nearly identical to the plasmid pMCR1_IncX4 (Accession no.: KU761327 from *K. pneumoniae* in China). It seems likely that pGD46-3 might be generated through the integration of the *mcr-1* gene into the specific site between the *pir* and *parA* genes of IncX4-type pSH146_32 plasmid (Additional file 2: Figure S5).

### Discovery of a new *mcr-1*-positive hybrid plasmid pGD17-2

The novel *mcr-1*-positive plasmid pGD17-2 we determined (Fig. 1 and Additional file 1: Table S1) allows the strain GD17 to grow well on the LBA plate supplemented with up to 16 mg/L of colistin (Additional file 2: Figure S9). The plasmid size of pGD17-2 is 121.454 kb (Additional file 1: Table S1), whose GC% is 43.69%. To rule out the possible errors in plasmid sequencing or assembly, hybrid plasmid pGD17-2 was further confirmed by subsequent PCR analysis and Sanger sequencing (Additional file 2: Figure S8 A and B). Comparative genomics revealed that pGD17-2 is a new hybrid one featuring with two types of plasmid backbones (IncI2 and IncFIB, in Fig. 1), implying temporary transition status of *mcr-1* transmission or genetic stability under special selection pressure. Relative to the paradigm *mcr-1*-harboring IncI2-type plasmid pHNSHP45, pGD17-2 exhibited 51% coverage and 99% identity and possessed two insertion sequences (IS*629* and IS*1294*) in the *nikB* gene, as well as an additional IS*629*-mediated insertion sequence (~56 kb) in the *pilO* gene (Fig. 1 and Additional file 2: Figure S2). Further sequence alignments suggested that the above insertion sequence (~56 kb) has only 26% coverage to a *mcr-1*-negative plasmid pHUSEC2011-2 (Accession no.: HE610901) (Fig. 1 and Additional file 2: Figure S2). Notably, the mobile genetic elements identified from the pGD17-2 plasmid are highly related to the cognate conjugation-associated counterparts or remnants (a syntenic F-like or R-like type IV secretion system gene clusters and a relaxase gene *traI* (Fig. 1 and Additional file 2: Figure S2). In comparison with all the *mcr-1*-carrying plasmids (Additional file 1: Table S3), pGD17-2 represents a new hybrid plasmid producing MCR-1 colistin resistance.

### Co-occurrence of two unique *mcr-1*-harboring plasmids (pGD65-3 and pGD65-4) in a single *E. coli* isolate

Unexpectedly, two *mcr-1-*bearing plasmids namely pGD65-3 and pGD65-4 (Fig. 2a and Additional file 2: Figure S1) were localized onto a single *E. coli* strain GD65 with significant colistin resistance (Additional file 2: Figure S9). The co-occurrence of these two plasmids was experimentally validated with Southern blot (Additional file 2: Figure S1B). Sequencing results showed that pGD65-3 (33.301 kb in length) is an IncI2-type plasmid, whereas the other one pGD65-4 (64.852 kb in length) is featuring a IncX4-type plasmid backbone. In similarity to all the other known IncI2-type plasmids, no insertion sequence IS*Apl1* is neighbored with the *mcr-1* gene in the pGD65-3 plasmid (Fig. 2b and Additional file 2: Figure S6). Although the IncX4-like plasmid pGD65-4 lacks the IS*Apl1* insertion sequence in front of the *mcr-1*, but it retains *pap2* at the 3′-end of the *mcr-1* locus (Fig. 2c, Additional file 2: Figures S4, S5 and S7). Conjugation-based PCR detection experiments demonstrated that the two plasmids (pGD65-3 and pGD65-4, in Fig. 2) can be separately transferred from the donor strain *E. coli* GD65 (Additional file 2: Figure S8C) to the recipient *E. coli* strain C600 (Additional file 2: Figure S8D) and give appreciable level of resistance to colistin (~16 μg/ml). Together, this result defined a paradigm for coexistence of two unique *mcr-1-*harboring plasmids (pGD65-4, IncX4 type, and pGD65-3, IncI2 type) in a single *E. coli* isolate.

## Conclusions

**Fig. 3:**
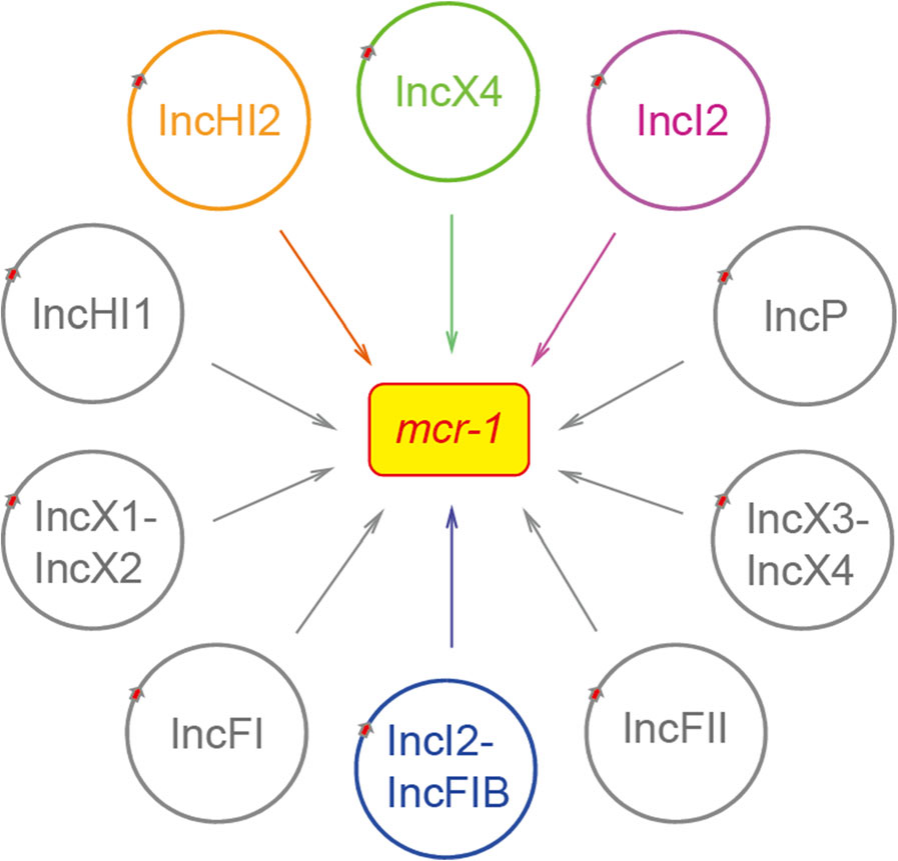
Scheme for the complexity in diversified *mcr-1*-bearing plasmids. The *mcr-1*-carrying plasmids are denoted with *circles*, in which the *mcr-1* gene is highlighted with *red arrow*. To the best of our knowledge, no less than ten families of plasmids produce the MCR-1 protein. Four types of *mcr-1*-containing plasmids are labeled in color, corresponding to IncX4 (*in green*), IncI2 (*in purple*), IncHI2 (*in orange*), and the hybrid version of IncI2-IncFIB (*in blue*), respectively

The data presented here extends landscapes of *mcr-1*-positive plasmids and highlights complexity of MCR-1 colistin resistance in gut microbiota (Fig. 3). Unlike the scenarios seen with the first reported *mcr-1*-harboring plasmid pHNSHP45, there are no IS*Apl1* element in front of the *mcr-1* gene in all the eight IncI2 plasmids we reported (pGD23-3, pGD53-3, pGD65-3, pGD81-1, pLishui142-1, pWH07-3, pWH09-3, and pWH13-4, in Additional file 2: Figure S7A). It is noted that all the IncI2 plasmids consistently contain a plasmid-borne site-specific recombinase gene, *rci* (Additional file 2: Figure S7A). Variations exist in the 3′-end sequence of *pap2* gene on IncI2 plasmids, of which the three plasmids (pWH07-3, pWH09-3, and pWH13-4) are almost the same and the other three plasmids (pGD23-3, pGD53-3 and pGD65-3) are highly identical (Additional file 2: Figure S7A). By contrast, the mosaic plasmid pGD17-2 is more flexible, with IS*629* and IS*1294* insertion in the *nikB* gene. And IS*Apl1* immediately appears in front of *mcr-1* in pGD17-2. Thus, the hybrid plasmid pGD17-2 represents a novel mobile element carrying *mcr-1* and provides remarkable insights into the plasmid-mediated transfer of MCR-1 colistin resistance.

In the *mcr-1*-harboring IncX4 plasmids (pGD46-3, pGD65-4, Lishui12, and pWH03), the regions between *parA* gene and *repA* gene are nearly identical (Additional file 2: Figure S7B), in that they shared 99% similarity to IncX4 *mcr-1*-harboring *K. pneumoniae* plasmid pMCR1_IncX4 (Accession no.: KU761327). In comparison with the IncX4 plasmid pSH146_32, the *mcr-1* gene was found to be inserted in the same location. Different with *mcr-1*-harboring IncI2 plasmids, the 3′-end sequences of *pap2* are consistently present in IncX4 plasmids. In addition, no IS*Apl1* elements were found to flank the *mcr-1* gene (Additional file 2: Figure S7B). The fact that strain GD65 carries two *mcr-1*-positive plasmids (pGD65-3 and pGD65-4) might represent a first example that both IncI2-type plasmid pGD65-3 and IncX4-type plasmid pGD65-4 contributed to colistin resistance in *E. coli*. Retrospectively, the two *mcr-1*-harboring plasmids (IncI2-like plasmid pHNSHP45 [6] and IncHI2-type plasmid pHNSHP45-2 [46]) from the *E. coli* strain SHP45 constitute an additional example. However, it seems likely the coexistence of two different *mcr-1*-positive plasmids does not confer distinguishable ability of the recipient strains in colistin resistance when compared with the strain with a single *mcr-1*-plasmid (Additional file 2: Figure S9). In general, two successive ATG codons initiate the *mcr-1* gene in most of the cases like GD17 and GD65. However, we noted that only one ATG in the case of strain Lishui142 (abbreviated as LS142). We therefore tested the function of this *mcr-1* variant [referred to *mcr-1*(Δ1)] using the pBAD24 expression system (Additional file 2: Figure S9). In fact, it gave indistinguishable ability when compared to the normal version *mcr-1* in the trials of colistin resistance, suggesting the redundancy of one “ATG” codon in the MCR-1.

In summary, no less than ten types of plasmids mediate the transmission of MCR-1 colistin resistance in field and clinical isolates (Fig. 3). Our results extended significantly our understanding of diversified *mcr-1*-bearing plasmids, whose dissemination constitutes a potential threat to public health and clinical treatment. Global distribution of *mcr-1* in *E. coli* populations emphasizes the importance to prevent global over- and/or mis-use of colistin. Extensive surveillance programs of antibiotic resistance should be extended to swine farms, and even in human medicine, since *mcr-1* has been recently identified in human isolates. Restrictive/rational use of colistin is urgently required to prevent the rapid spread of *mcr-1* to other bacteria and in different niches, excluding human hospitals and foodborne settings.

## Additional files

Additional file 1: Table S1. New *mcr-1*-harboring plasmids with full genomes sequenced in this study. **Table S2.** Primers used in this study. **Table S3.** List of *mcr-1*-positive plasmids with known genomes. (DOC 237 kb)

Additional file 2: Figure S1. Genetic evidence for the diversified *mcr-1*-positive plasmids. **Figure S2.** Comparative genomics of the eight IncI2 type *mcr-1*-carrying plasmids. **Figure S3.** Genome comparison of the pGD65-3 plasmid with the *mcr-1*-carrying plasmid pHNSHP45-2. **Figure S4.** Co-linear genome alignments for the four IncX4 type *mcr-1*-carrying plasmids. **Figure S5.** Genomic comparison of the newly determined plasmid pGD46-3 with the recently reported one pmcr-1_IncX4 and the *mcr-1*-lacking plasmid pSH146_32. **Figure S6.** Genomic analyses for the three IncI2-type plasmids. **Figure S7.** Fine mapping of the *mcr-1*-surrounding regions. **Figure S8.** Genetic identification and characterization of the fourteen *mcr-1*-harboring plasmids. **Figure S9.** Measurement of the ability of clinical (and/or engineered) *E. coli* strains in colistin resistance. (DOC 3018 kb)

## Abbreviations

*A. baumannii*: *Acinetobacter baumannii*
ESBL: Extended spectrum β-lactamases
GalN: Galactosamine
*K. pneumoniae*: *Klebsiella pneumoniae*
*mcr-1*: Mobilized colistin resistance gene
MDR: Multidrug-resistant
NDM-1: New Delhi β-lactamase 1
*P. aeruginosa*: *Pseudomonas aeruginosa*
pEtN: Phosphoethanolamine
*S. enterica*: *Salmonella enterica*
