# Supplementary material for: Expanding landscapes of the diversified mcr-1-bearing plasmid reservoirs
Source: Microbiome. 2017 Jul 6;5:70. doi: 10.1186/s40168-017-0288-0 (PMC5500976; doi:10.1186/s40168-017-0288-0)
Supplement: Supplementary file 1 — New mcr-1-harboring plasmids with full genomes sequenced in this study. Table S2. Primers used in this study. Table S3. List of mcr-1-positive plasmids with known genomes. (DOC 237 kb) [file 40168_2017_288_MOESM1_ESM.doc]

**Additional file Tables**

**Table S1** New *mcr-1*-harbouring plasmids with full genomes sequenced in this study

| **Plasmids** | **Size (bp)** | **Inc-type** | ***E. coli*** | **Time** | **Acc. no.** |
| --- | --- | --- | --- | --- | --- |
| **pGD17-2** | 121,450 | IncFIB | Swine isolate | 2016.1 | KY075650 |
|  |  | IncI2 |  |  |  |
| **pGD65-3** | 64,852 | IncI2 | Swine isolate | 2016.1 | KY075661 |
| **pGD65-4** | 33,301 | IncX4 |  |  | KY075660 |
| pGD80-2 | 241,031 | IncHI2 | Swine isolate | 2016.1 | KY075659 |
| pGD23-3 | 64,074 | IncI2 | Swine isolate | 2016.1 | KY075651 |
| pGD53-3 | 62,813 |  | Swine isolate | 2016.1 | KY075662 |
| pLS142-1 | 61,906 |  | Human isolate | 2016.1 | KY075654[^1^](#_ENREF_1) |
| pWH07-3 | 62,066 |  | Swine isolate | 2011.8 | KY075658 |
| pWH09-3 | 62,072 |  | Swine isolate | 2011.8 | KY075657 |
| pWH13-4 | 60,693 |  | Swine isolate | 2012.6 | KY075656 |
| pGD81-1 | 62,712 |  | Swine isolate | 2016.1 | KY446064 |
| pLS12-2 | 33,307 | IncX4 | Human isolate | 2016.1 | KY075653[^1^](#_ENREF_1) |
| pGD46-3 | 33,298 |  | Swine isolate | 2016.1 | KY075652 |
| pWH03-3 | 33,251 |  | Swine isolate | 2011.8 | KY075655 |

**Table S2** Primers used in this study

| **Primers** | **Position** (bp) | **Primer sequence** (5’-3’) |
| --- | --- | --- |
| hpGD17-F1 | 10927--10947 | GTCCCCTGGAATATTTCTCGT |
| hpGD17-R1 | 13099--13119 | TTGTTCATCTTTATGCGGGTC |
| hpGD17-F2 | 64521--64541 | TCCAGGCTTTAACTACGTCCA |
| hpGD17-R2 | 67942--67960 | ACCGGAAAACCAGTAAGCA |
| clsGD17-F | 30138--30159 | CACCATAACTAAGTTTGCAGGG |
| clsGD17-R | 31025--31049 | GTATGTAATTAATCAACAGACAAGG |
| clsGD23-F | 30459--30483 | CCTGTGTAACTCTCAATTAAGTTTG |
| clsGD23-R | 29904--29926 | GGTGAGTACCGCCATAAAAGTTG |
| clsGD46-F | 8127--8150 | AGGTTGAATGCATTTATCGAAGAG |
| clsGD46-R | 8647--8670 | CTTTTTCTTTTCGCTTACCGTTAC |
| clsGD53-F | 29535--29554 | GTGCCAATCCGGTCGGTGGT |
| clsGD53-R | 30949--30971 | GCCAGTGACGATACTTATCACTG |
| clsGD65-3-F | 31176--31197 | GAACTTCACTACCAGTACGTAC |
| clsGD65-3-R | 32085--32106 | GTACCTGGAGTGTCAGTAATGC |
| clsGD65-4-F | 27747--27769 | CACTAAAAGTTGCAGACATTGTG |
| clsGD65-4-R | 28266--28288 | CCAGTTTTGTTTTGTTCTCTGTG |
| clsGD80-F | 92311--92331 | CCGCTGTGAAGGGTATAACCC |
| clsGD80-R | 93226--93248 | CATAAATTTGCATGGCGTTCCGG |
| clsGD81-F | 62297--62320 | GTCAATTAAAGCAACGATACAATG |
| clsGD81-R | 402--424 | CATTCCATTGATAGATCCTGACG |
| clsLS12-F | 14013--14037 | GTTCTTATAGCTAAAGGTTTTGCTG |
| clsLS12-R | 14527--14551 | CAAAATAATGAAGCATGTCACAACC |
| clsLS142-F | 34936--34957 | AATCAGGTGTGTAAGAGCACGC |
| clsLS142-R | 35891--35911 | CAGGGTCCGGTAAGTCAACGA |
| clsWH03-F | 26973--26995 | GATAAAGGCGGTACAGGTAAATC |
| clsWH03-R | 27493--27514 | CCCATTCCCTGATTCATTGCTC |
| clsWH07-F | 58409--58432 | CAGTATTTGAGACTGTTTTTCCCC |
| clsWH07-R | 59144--59163 | GTGGAAATGCCGCCGATGCG |
| clsWH09-F | 33366--33389 | CAATAACACTTCCATCAGTGTTGC |
| clsWH09-R | 34092--34115 | GAAAGAACGCTGGACAAGTTCTAT |
| clsWH13-F | 60210--60231 | GGTTTCCTTTGGCACGTTGGGA |
| clsWH13-R | 473--494 | GCGCTTTTCAGTGATGGGTTGG |

Designations: hp, hybrid plasmid; cls, close-loop sequence

**Table S3** List of *mcr-1*-positive plasmids with known genomes

| **No.** | **Plasmids** | **Hosts** | **Size (bp)** | **Inc-type** | **Acc. No.** | **Ref.** |
| --- | --- | --- | --- | --- | --- | --- |
| 1 | pHNSHP45 | *E. coli* | 64,015 | IncI2 | KP347127 | [^2^](#_ENREF_2) |
| 2 | pMCR1_IncI2 | *E. coli* | 64,964 |  | KU761326 | [^3^](#_ENREF_3) |
| 3 | pABC149-MCR-1 | *E. coli* | 61,228 |  | KX013538 | [^4^](#_ENREF_4) |
| 4 | pVT553 | *E. coli* | 62,218 |  | KU870627 | [^5^](#_ENREF_5) |
| 5 | pBA76-MCR-1 | *E. coli* | 64,942 |  | KX013540 | [^4^](#_ENREF_4) |
| 6 | pBA77-MCR-1 | *E. coli* | 62,661 |  | KX013539 | [^4^](#_ENREF_4) |
| 7 | pAf23 | *E. coli* | 61,177 |  | KX032519 | [^6^](#_ENREF_6) |
| 8 | pA31-12 | *E. coli* | 67,134 |  | KX034083 | [^7^](#_ENREF_7) |
| 9 | pEG430-1 | *Shigella sonnei* | 61,826 |  | LT174530 | [^8^](#_ENREF_8) |
| 10 | pSCS23 | *S. enterica* | 65,419 |  | KU934209 | [^9^](#_ENREF_9) |
| 11 | pECJS-61-63 | *E. coli* | 63,656 |  | KX254342 | / |
| 12 | pECJP-61-63 | *E. coli* | 63,656 |  | KX084393 | / |
| 13 | pMCR_1410 | *Kluyvera ascorbata* | 57,059 |  | KU922754 | / |
| 14 | pS2.14-2 | *E. coli* | 60,950 |  | CP016187 | / |
| 15 | pEC13-1 | *E. coli* | 60,218 |  | CP016186 | / |
| 16 | pEC5-1 | *E. coli* | 61,735 |  | CP016185 | / |
| 17 | pSLy1 | *E. coli* | 65,888 |  | CP015913 | / |
| 18 | pSLy21 | *E. coli* | 63,329 |  | CP016405 | / |
| 19 | pESTMCR | *E. coli* | 33,311 | IncX4 | KU743383 | [^10^](#_ENREF_10) |
| 20 | pOW3E1 | *E. coli* | 34.640 |  | KX129783 | [^11^](#_ENREF_11) |
| 21 | pAf48 | *E. coli* | 31,808 |  | KX032520 | [^6^](#_ENREF_6) |
| 22 | pKP37-BE | *E. coli* | 35,104 |  | LT598652 | [^12^](#_ENREF_12) |
| 23 | pMCR1_IncX4 | *K. pneumoniae* | 33,287 |  | KU761327 | [^3^](#_ENREF_3) |
| 24 | pMCR1.2-IT | *K. pneumoniae* | 33,303 |  | KX236309 | [^13^](#_ENREF_13) |
| 25 | pICBEC72Hmcr | *E. coli* | 33,304 |  | CP015977 | [^14^](#_ENREF_14) |
| 26 | pMCR1-NJ-IncX4 | *E. coli* | 33,395 |  | KX447768 | [^15^](#_ENREF_15) |
| 27 | pECGD-8-33 | *E. coli* | 33,307 |  | KX254343 | / |
| 28 | pMCR1-NJ-IncX4 | *E. coli* | 33,395 |  | KX447768 | / |
| 29 | pECJP-B65-33 | *E. coli* | 33,298 |  | KX084392 | / |
| 30 | unnamed4 | *E. coli* | 49,695 |  | CP016550 | / |
| 31 | pICBEC7Pmcr | *E. coli* | 34,992 |  | CP017246 | / |
| 32 | pKH457-3-BE | *E. coli* | 79,798 | IncP | KU353730 | [^16^](#_ENREF_16) |
| 33 | pCQ02-121 | *E. coli* | 48,350 | IncN | KU647721 | / |
| 34 | pMR0516mcr | *E. coli* | 225,707 | IncF | KX276657 | [^17^](#_ENREF_17) |
| 35 | pKP81-BE | *E. coli* | 91,041 | IncFII | KU994859 | [^18^](#_ENREF_18) |
| 36 | pH226B | *E. coli* | 209,401 | IncHI1 | KX129784 | [^11^](#_ENREF_11) |
| 37 | pHNSHP45-2 | *E. coli* | 251,493 | IncHI2 | KU341381 | [^19^](#_ENREF_19) |
| 38 | pS38 | *E. coli* | 247,885 |  | KX129782 | [^11^](#_ENREF_11) |
| 39 | pSA26-MCR-1 | *E. coli* | 240,367 |  | KU743384 | [^4^](#_ENREF_4) |
| 40 | pECJS-B60-267 | *E. coli* | 267,486 |  | KX254341 | / |
| 41 | pECJP-59-244 | *E. coli* | 243,572 | IncHI2 | KX084394 | / |
| 42 | pEC2-4 | *E. coli* | 235,403 | IncHI1A | CP016184 | / |
| 43 | pEC2_1-4 | *E. coli* | 230,278 | IncHI1A | CP016183 | / |
| 44 | p100R | *E. coli* | 26,403 | / | KX090925 | / |
| 45 | unnamed1 | *E. coli* | 15,998 | / | KX528699 | / |
| 46 | pHNSH36 | *S. enterica* | 54,502 | / | KX257480 | / |
| 47 | pHNSH138 | *S. enterica* | 54,670 | / | KX257481 | / |
| 48 | pHNZ319S | *S. enterica* | 54,494 | / | KX257482 | / |

/, not detected/available

The sequence data is retrieved from the NCBI database (<https://ftp.ncbi.nlm.nih.gov/genbank)> as of Oct 28, 2016.

**Additional file references**

1 Wang, Q. *et al.* Genomic insights into *mcr-1*-positive plasmids carried by the colistin-resistant *Escherichia coli* from the inpatients. *Antimicrobial agents and chemotherapy*, doi:10.1128/AAC.00361-17 (2017).

2 Liu, Y. Y. *et al.* Emergence of plasmid-mediated colistin resistance mechanism MCR-1 in animals and human beings in China: a microbiological and molecular biological study. *Lancet Infect Dis* **16**, 161-168, doi:10.1016/S1473-3099(15)00424-7 (2016).

3 Li, A. *et al.* Complete sequences of *mcr-1*-harboring plasmids from extended-spectrum-beta-lactamase- and carbapenemase-producing Enterobacteriaceae. *Antimicrobial agents and chemotherapy* **60**, 4351-4354, doi:10.1128/AAC.00550-16 (2016).

4 Sonnevend, A. *et al.* Plasmid-mediated colistin resistance in Escherichia coli from the Arabian Peninsula. *International journal of infectious diseases : IJID : official publication of the International Society for Infectious Diseases*, doi:10.1016/j.ijid.2016.07.007 (2016).

5 Perreten, V., Strauss, C., Collaud, A. & Gerber, D. Colistin Resistance Gene mcr-1 in Avian-Pathogenic Escherichia coli in South Africa. *Antimicrobial agents and chemotherapy* **60**, 4414-4415, doi:10.1128/AAC.00548-16 (2016).

6 Poirel, L. *et al.* Genetic Features of MCR-1-Producing Colistin-Resistant Escherichia coli Isolates in South Africa. *Antimicrobial agents and chemotherapy* **60**, 4394-4397, doi:10.1128/AAC.00444-16 (2016).

7 Sun, J. *et al.* Complete nucleotide sequence of an IncI2 plasmid coharboring *bla*CTX-M-55 and *mcr-1*. *Antimicrobial agents and chemotherapy* **60**, 5014-5017, doi:10.1128/AAC.00774-16 (2016).

8 Pham Thanh, D. *et al.* Inducible colistin resistance via a disrupted plasmid-borne mcr-1 gene in a 2008 Vietnamese Shigella sonnei isolate. *The Journal of antimicrobial chemotherapy* **71**, 2314-2317, doi:10.1093/jac/dkw173 (2016).

9 Yang, Y. Q. *et al.* Co-occurrence of *mcr-1* and ESBL on a single plasmid in *Salmonella enterica*. *The Journal of antimicrobial chemotherapy* **71**, 2336-2338, doi:10.1093/jac/dkw243 (2016).

10 Brauer, A. *et al.* Plasmid with colistin resistance gene mcr-1 in ESBL-producing Escherichia coli strains isolated from pig slurry in Estonia. *Antimicrobial agents and chemotherapy*, doi:10.1128/AAC.00443-16 (2016).

11 Zurfluh, K., Klumpp, J., Nuesch-Inderbinen, M. & Stephan, R. Full-Length Nucleotide Sequences of mcr-1-Harboring Plasmids Isolated from Extended-Spectrum-beta-Lactamase-Producing Escherichia coli Isolates of Different Origins. *Antimicrobial agents and chemotherapy* **60**, 5589-5591, doi:10.1128/AAC.00935-16 (2016).

12 Xavier, B. B. *et al.* Identification of a novel plasmid-mediated colistin-resistance gene, mcr-2, in Escherichia coli, Belgium, June 2016. *Euro surveillance : bulletin Europeen sur les maladies transmissibles = European communicable disease bulletin* **21**, doi:10.2807/1560-7917.ES.2016.21.27.30280 (2016).

13 Di Pilato, V. *et al.* mcr-1.2, a New mcr Variant Carried on a Transferable Plasmid from a Colistin-Resistant KPC Carbapenemase-Producing Klebsiella pneumoniae Strain of Sequence Type 512. *Antimicrobial agents and chemotherapy* **60**, 5612-5615, doi:10.1128/AAC.01075-16 (2016).

14 Fernandes, M. R. *et al.* First report of the globally disseminated IncX4 plasmid carrying the *mcr-1* gene in a colistin-resistant *Escherichia coli* sequence type 101 isolate from a human infection in Brazil. *Antimicrobial agents and chemotherapy* **60**, 6415-6417, doi:10.1128/AAC.01325-16 (2016).

15 Mediavilla, J. R. *et al.* Colistin- and carbapenem-resistant *Escherichia coli* harboring *mcr-1* and *bla*NDM-5, causing a complicated urinary tract infection in a patient from the United States. *mBio* **7**, doi:10.1128/mBio.01191-16 (2016).

16 Malhotra-Kumar, S. *et al.* Colistin resistance gene mcr-1 harboured on a multidrug resistant plasmid. *The Lancet. Infectious diseases* **16**, 283-284, doi:10.1016/S1473-3099(16)00012-8 (2016).

17 McGann, P. *et al.* *Escherichia coli* harboring *mcr-1* and *bla*CTX-M on a novel IncF plasmid: First report of *mcr-1* in the United States. *Antimicrobial agents and chemotherapy* **60**, 4420-4421, doi:10.1128/AAC.01103-16 (2016).

18 Xavier, B. B., Lammens, C., Butaye, P., Goossens, H. & Malhotra-Kumar, S. Complete sequence of an IncFII plasmid harbouring the colistin resistance gene *mcr-1* isolated from Belgian pig farms. *The Journal of antimicrobial chemotherapy* **71**, 2342-2344, doi:10.1093/jac/dkw191 (2016).

19 Zhi, C., Lv, L., Yu, L. F., Doi, Y. & Liu, J. H. Dissemination of the *mcr-1* colistin resistance gene. *Lancet Infect Dis* **16**, 292-293, doi:10.1016/S1473-3099(16)00063-3 (2016).
