# Supplementary material for: Expanding landscapes of the diversified mcr-1-bearing plasmid reservoirs
Source: Microbiome. 2017 Jul 6;5:70. doi: 10.1186/s40168-017-0288-0 (PMC5500976; doi:10.1186/s40168-017-0288-0)
Supplement: Supplementary file 2 — Genetic evidence for the diversified mcr-1-positive plasmids. Figure S2. Comparative genomics of the eight IncI2 type mcr-1-carrying plasmids. Figure S3. Genome comparison of the pGD65-3 plasmid with the mcr-1-carrying plasmid pHNSHP45-2. Figure S4. Co-linear genome alignments for the four IncX4 type mcr-1-carrying plasmids. Figure S5. Genomic comparison of the newly determined plasmid pGD46-3 with the recently reported one pmcr-1_IncX4 and the mcr-1-lacking plasmid pSH146_32. Figure S6. Genomic analyses for the three IncI2-type plasmids. Figure S7. Fine mapping of the mcr-1-surrounding regions. Figure S8. Genetic identification and characterization of the fourteen mcr-1-harboring plasmids. Figure S9. Measurement of the ability of clinical (and/or engineered) E. coli strains in colistin resistance. (DOC 3018 kb) [file 40168_2017_288_MOESM2_ESM.doc]

**Additional file figures**


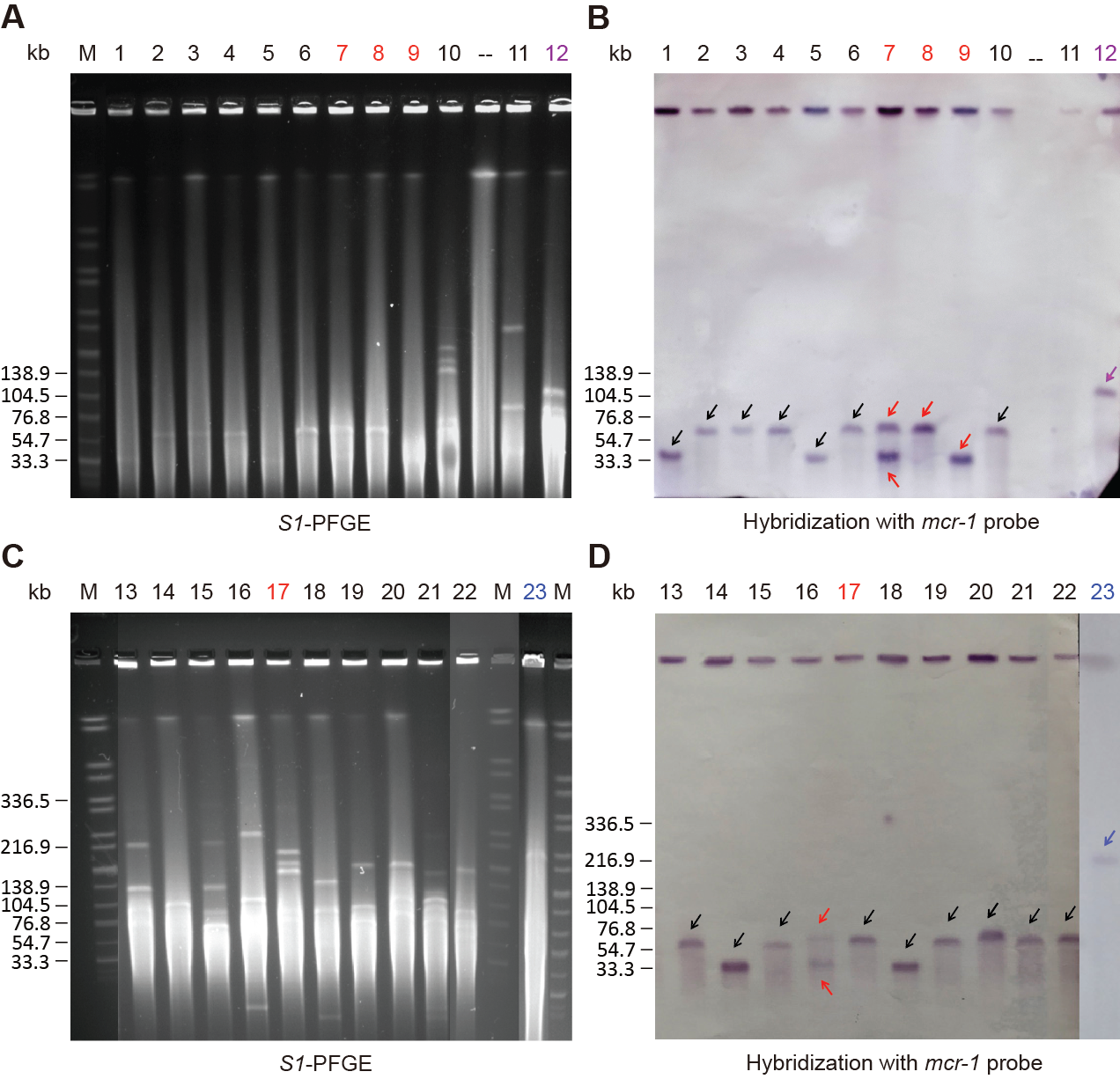


**Figure S1** Genetic evidence for the diversified *mcr-1*-positive plasmids

**A.** S1-PFGE analyses of the trans-conjugant strains with colistin resistance

**B.** Southern blot analyses of the plasmids with the specific *mcr-1* probe

The lanes (labeling from 1, 2, 3, …, to 12) referred to trans-conjugant strains [WH03T, WH07T, WH09T, WH13T, GD46T, GD53T, GD65-3,4T, GD65-3T, GD65-4T, GD81T, Lishui12, and GD17]. Minus denotes negative control strain *E. coli* C600.

**C.** S1-PFGE assays for the clinical *E. coli* isolate with colistin resistance

**D.** Southern blot analyses of the plasmids with the specific *mcr-1* probe

The numbers labeled from 13, 14, 15, …, to 23 (in **panel C** & **D**) indicate strains GD23, GD46, GD53, GD65, GD81, WH03, WH07, WH09, WH13, Lishui142, and GD80, respectively. “M” denotes the DNA marker that is the XbaI-digested genomic DNA from the reference strain H9812 of *Salmonella enterica*.

The *mcr-1*-containing DNA fragments are highlighted with arrows (**B** & **D**).

Two plasmids carried by the strain GD65 are indicated with red arrows, the hybrid plasmid from strain GD17 is shown with a purple arrow (**B**), and the mega plasmid (~240 kb) in the strain GD80 is highlighted in blue (**D**).


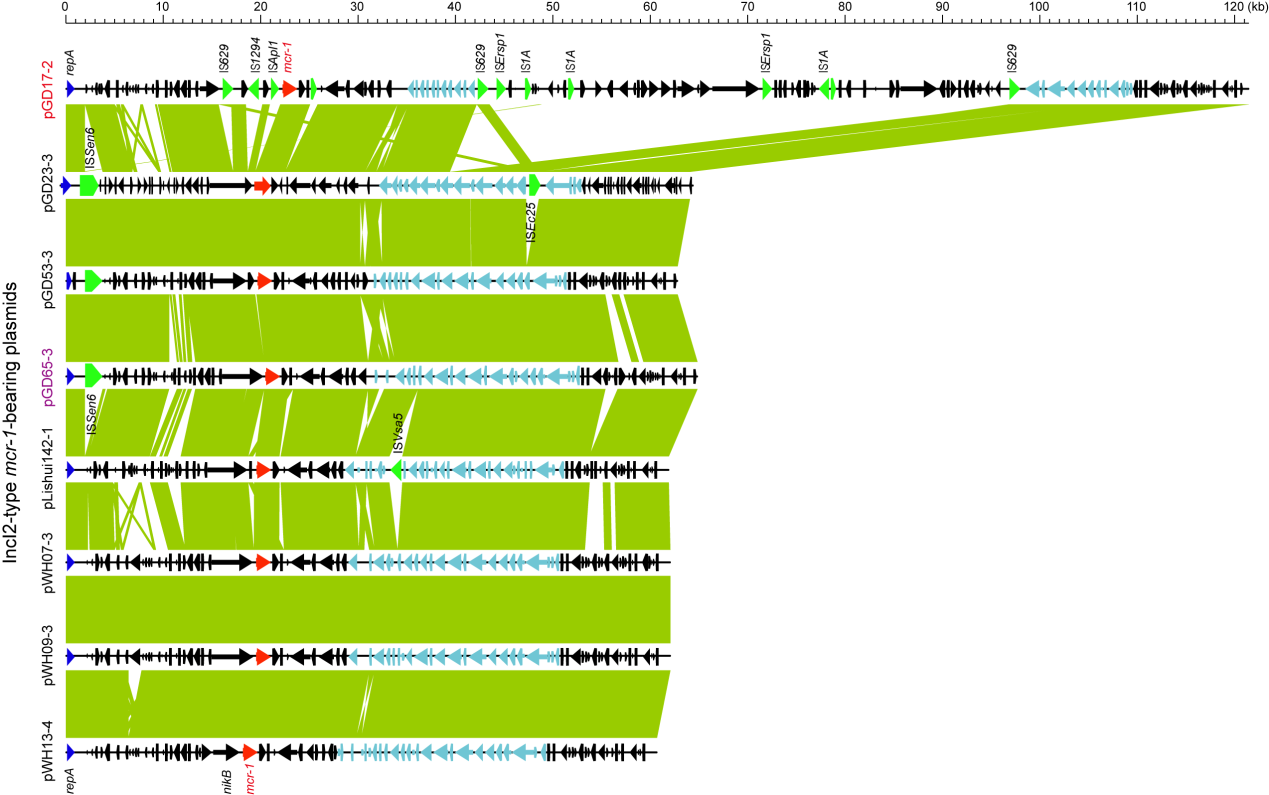


**Figure S2** Comparative genomics of the eight IncI2 type *mcr-1*-carrying plasmids

The eight *mcr-1*-positive plasmids whose full genome sequences were decoded in our study correspond to pGD17-2, pGD23-3, pGD53-3, pGD65-3, pLishui142-1, pWH07-3, pWH09-3, and pWH13-4, respectively (**Table 1**). The plasmid pGD17-2 is indicated in red, whereas the plasmid pGD65-3 is highlighted in purple.

The schematic diagram is drawn to scale, and the region (>99% homology) is shaded in green. Genes associated with the *tra* and *pil* loci are denoted with light blue arrows, while replication-associated genes are highlighted in dark blue arrows. The *mcr-1* gene is represented with the red arrow, while accessory genes are expressed with black arrows. Insertion sequences are highlighted in green arrows.


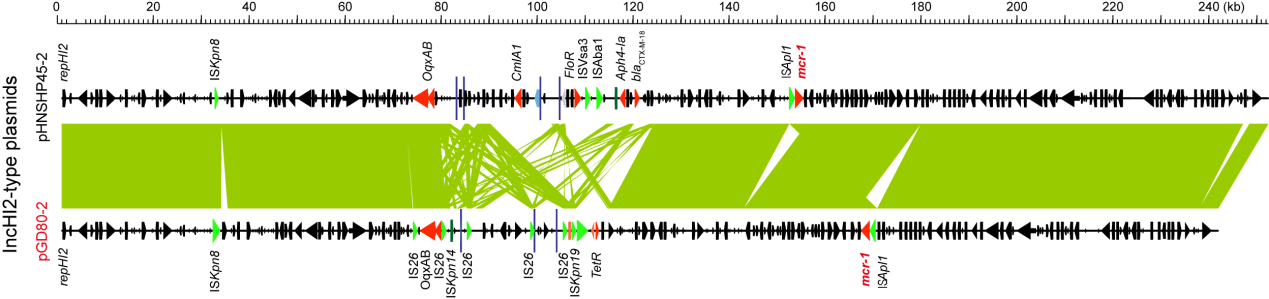


**Figure S3** Genome comparison of the pGD65-3 plasmid with the *mcr-1*-carrying plasmid pHNSHP45-2

Of note, the two *mcr-1*-harbouring plasmids (pGD65-3 and pHNSHP45-2) here are assigned into the members of the IncHI2 family.


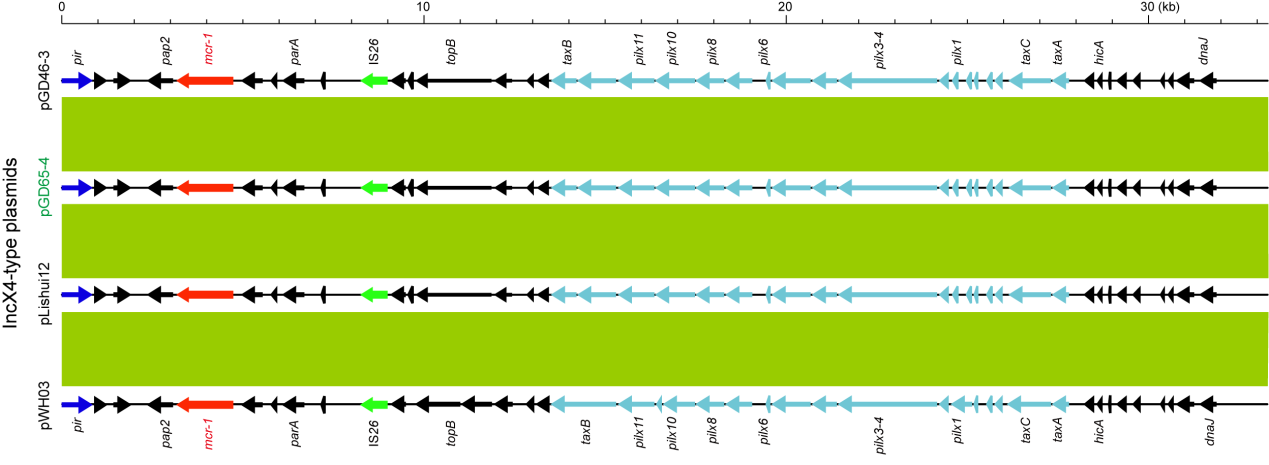


**Figure S4** Co-linear genome alignments for the four IncX4 type *mcr-1*-carrying plasmids

The four *mcr-1*-bearing plasmids with full genome sequences decoded in this study included pGD46-3, pGD65-4, pLishui12, and pWH03, respectively (**Table 1**). The plasmid pGD65-4 is highlighted in green.


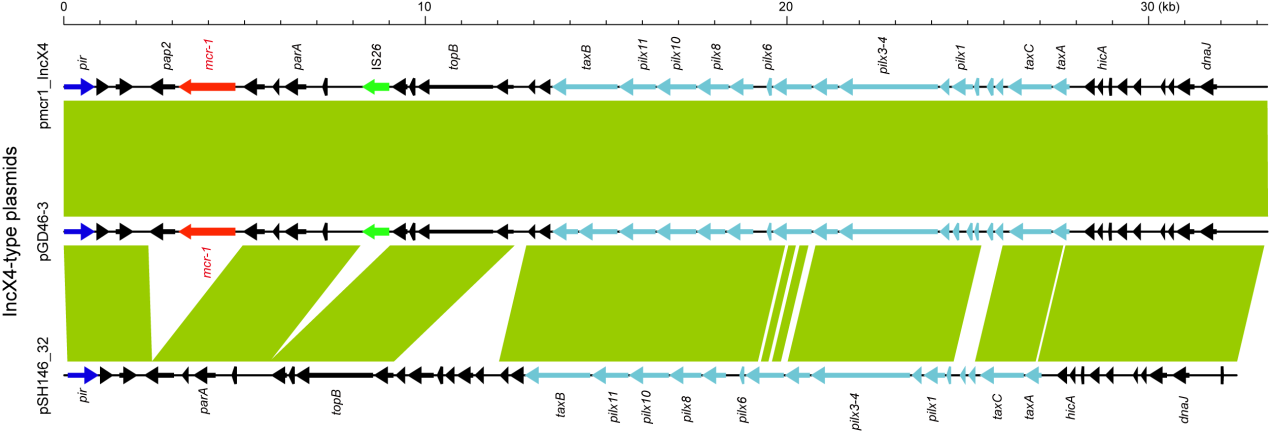


**Figure S5** Genomic comparison of the newly-determined plasmid pGD46-3 with the recently-reported one pmcr-1_IncX4 and the *mcr-1*-lacking plasmid pSH146_32)

Of note, the above three plasmids belong to members of the IncX4-type plasmid family.


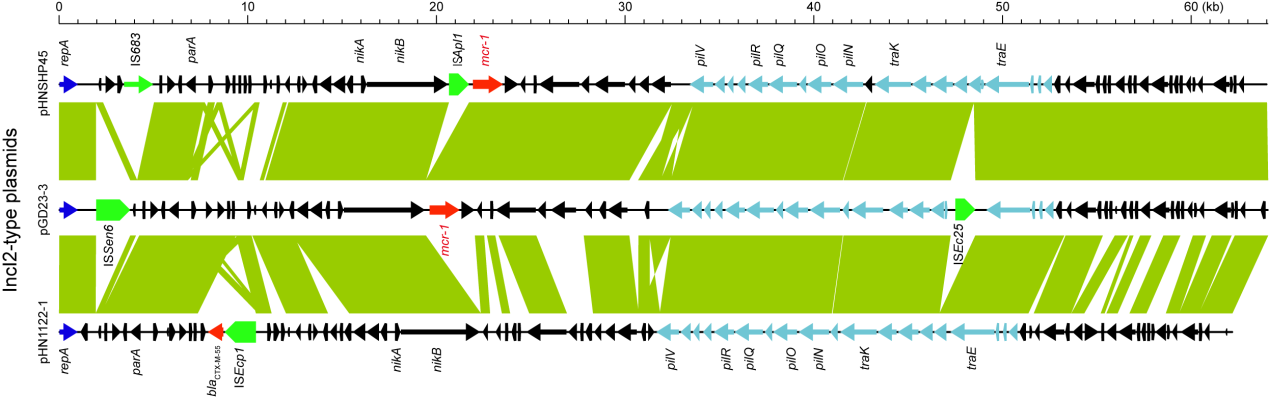


**Figure S6** Genomic analyses for the three IncI2-type plasmids

The two *mcr-1*-positive plasmids denote pGD23_3 we determined in this study, and pHNSHP45, a paradigm version of *mcr-1*-harbouring plasmid. In contrast, the plasmid pHN1122-1 is an ancestor for plasmid background without the *mcr-1* gene.


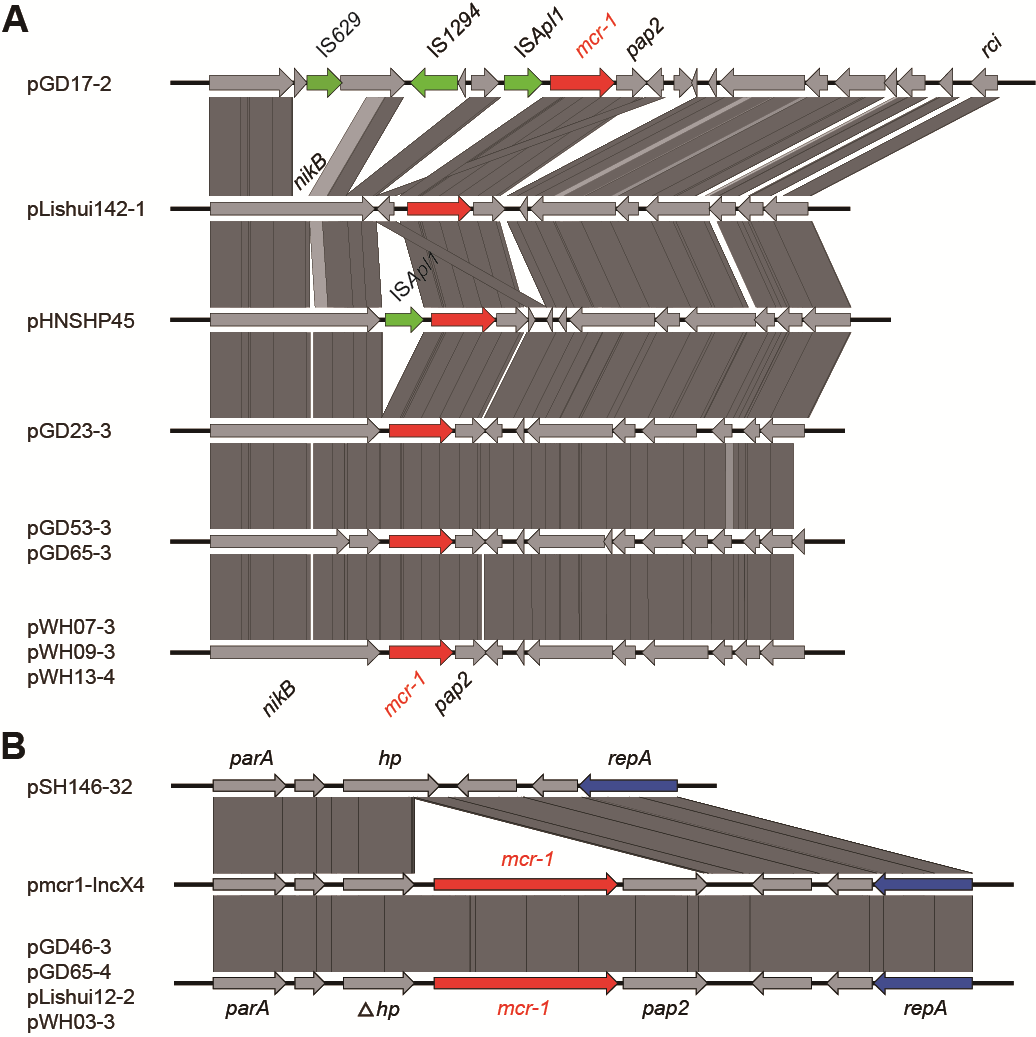


**Figure S7** Fine mapping of the *mcr-1*-surrouding regions

**A.** Schematic representations of the genetic organization surrounding *mcr-1* on the nine IncI2 plasmids

The nine *mcr-1*-bearing plasmids denote pGD17-2, pLishui142-1, pHNSHP45 (accession number KP347127.1), pGD23-3, pGD53-3, pGD65-3, pWH07-3, pWH09-3, and pWH13-4, respectively.

**B.** Schematic representations of the genetic organization surrounding *mcr-1* on the six IncX4 plasmids

The six IncX4 plasmids separately included pSH146-32, pmcr1-IncX4 (accession number KU761327.1), pGD46-3, pGD65-4, pLishui12-2 and pWH03-3.

Annotation is identical to that of **Figure S1**.


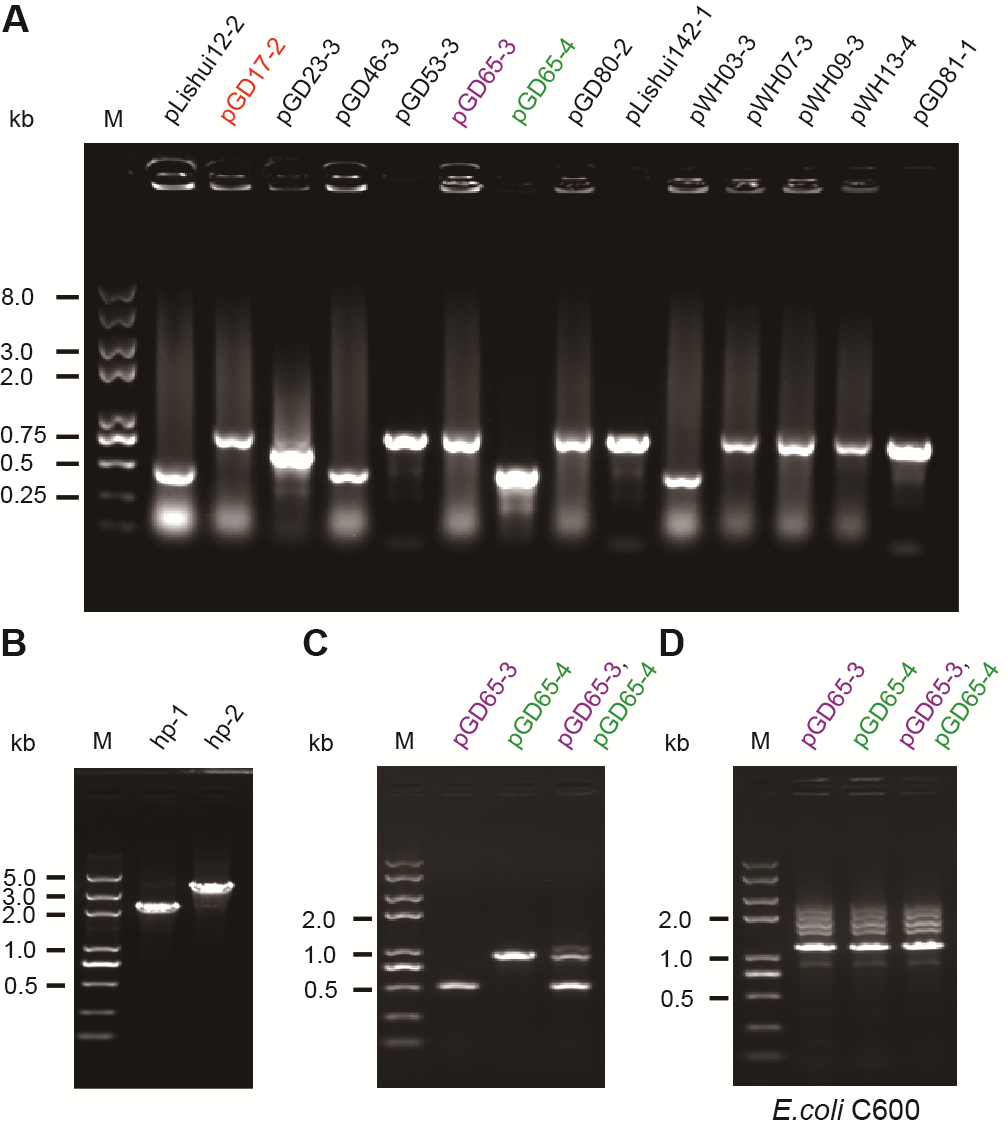


**Figure S8** Genetic identification and characterization of the fourteen *mcr-1*-harbouring plasmids

**A.** PCR-based close-loop analyses for the 14 plasmids carrying the *mcr-1* gene

The primers used for close-loop PCR trials are listed in **Table S2**.

**B.** PCR assay for the IncI2/IncFIB heterogeneity in the new hybrid plasmid pGD17-2

The two specific PCR products (*hp-1*, 2192 bp and *hp-2*, 3439 bp) are given in this assay.

The two co-existing plasmids (pGD65-3 and pGD65-4) can be transferred from the *E. coli* strain GD65 (**C**) to the same host *E. coli* C600 by conjugation (**D**).


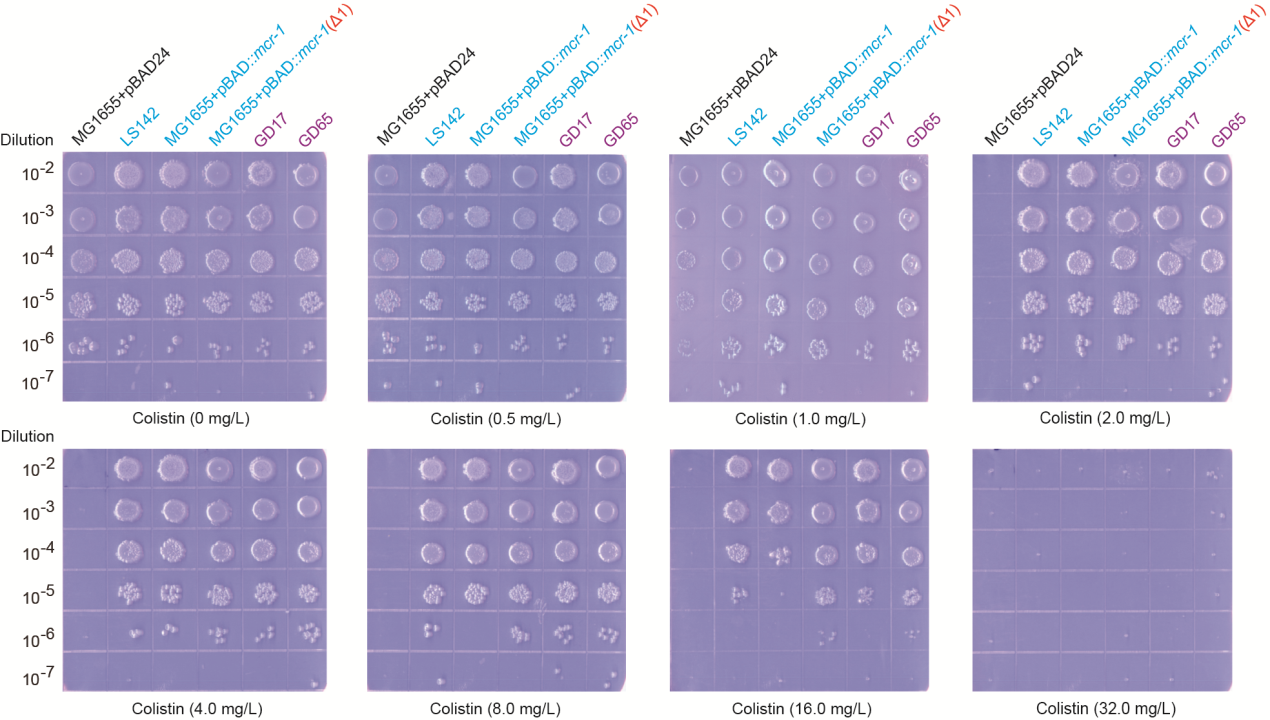


**Figure S9** Measurement of the ability of clinical (and/or engineered) *E. coli* strains in colistin resistance

The three clinical strains of *E. coli* included LS142, GD17, and GD65, respectively, whereas the engineered strains referred to MG1655 with plasmid pBAD24-driven expression of the *mcr-1* and/or its derivative. The strain MG1655 with the pBAD24 empty vector serves as the negative control. The level of colistin resistance was determined with the method of plating in series of dilution. In brief, the log phase cultures (OD600 = ~1.0) in serial dilution were spotted on LBA plates with different level of colistin (0, 0.5, 1.0, 2.0, 4.0, 8.0, 16.0 and 32.0 mg/L) and 0.2% arabinose. The LBA plates were maintained overnight at 37^o^C.

In general, two ATG codons appear at the initial position of the *mcr-1* gene in most of cases like GD17 and GD65. However, we noted that only one ATG in the case of LS142 strain. We therefore tested its function of this *mcr-1* variant [referred to *mcr-1*(Δ1)] using the pBAD24 expression system. In fact, it gave indistinghishable ability whem comapred to the normal version *mcr-1* in the trials of colistin resistance.
